# Functioning of Fluorescent Proteins in Aggregates in Anthozoa Species and in Recombinant Artificial Models

**DOI:** 10.3390/ijms18071503

**Published:** 2017-07-12

**Authors:** Natalia V. Povarova, Natalia D. Petri, Anna E. Blokhina, Alexey M. Bogdanov, Nadya G. Gurskaya, Konstantin A. Lukyanov

**Affiliations:** 1 Shemyakin-Ovchinnikov Institute of Bioorganic Chemistry, Miklukho-Maklaya 16/10, 117997 Moscow, Russia; povarovanv@gmail.com (N.V.P.); petri1543@inbox.ru (N.D.P.); eonornia@ya.ru (A.E.B.); noobissat@gmail.com (A.M.B.); ngurskaya@mail.ru (N.G.G.); 2 Nizhny Novgorod State Medical Academy, 10/1 Minin and Pozharsky Sq., 603005 Nizhny Novgorod, Russia

**Keywords:** green fluorescent protein (GFP), red fluorescent protein (RFP), FRAP, aggregation, gene expression marker

## Abstract

Despite great advances in practical applications of fluorescent proteins (FPs), their natural function is poorly understood. FPs display complex spatio-temporal expression patterns in living Anthozoa coral polyps. Here we applied confocal microscopy, specifically, the fluorescence recovery after photobleaching (FRAP) technique to analyze intracellular localization and mobility of endogenous FPs in live tissues. We observed three distinct types of protein distributions in living tissues. One type of distribution, characteristic for *Anemonia*, *Discosoma* and *Zoanthus*, is free, highly mobile cytoplasmic localization. Another pattern is seen in FPs localized to numerous intracellular vesicles, observed in *Clavularia*. The third most intriguing type of intracellular localization is with respect to the spindle-shaped aggregates and lozenge crystals several micrometers in size observed in *Zoanthus* samples. No protein mobility within those structures was detected by FRAP. This finding encouraged us to develop artificial aggregating FPs. We constructed “trio-FPs” consisting of three tandem copies of tetrameric FPs and demonstrated that they form multiple bright foci upon expression in mammalian cells. High brightness of the aggregates is advantageous for early detection of weak promoter activities. Simultaneously, larger aggregates can induce significant cytostatic and cytotoxic effects and thus such tags are not suitable for long-term and high-level expression.

## 1. Introduction

To date, proteins of the green fluorescent protein (GFP) family have been found in species of Hydrozoa, Anthozoa, Copepoda, and Cephalochordata [1,2,3,4]. Such dispersed distribution throughout distant taxa suggests that GFP-like proteins originated at very early stages of evolution of animal kingdom [3]. GFP-like proteins display different colors, the most common among the natural proteins being green, but also cyan, yellow, orange-red fluorescent, as well as non-fluorescent colors of various hues. GFP-like proteins from Anthozoa species possess the widest color diversity compared to other groups; a particular specimen typically expresses several proteins of different colors [2,5,6].

Fluorescent proteins (FPs) represent an indispensable tool in modern biology, enabling protein and cell labeling in live models [7]. Practical interest in development of new improved FPs and FP-based methods has stimulated extensive research on FP structure, biochemistry, photochemistry, and photophysics. At the same time, the biological role of FPs remains less well understood. Recent experimental studies have provided support for a sunscreening function of red fluorescent proteins and nonfluorescent purple-blue chromoproteins in symbiotic shallow water corals [8,9]. However, the high-level expression of fluorescent proteins in corals from mesophotic habitats in which an undersupply rather than an oversupply of light challenges the performance of symbiotic corals suggests that the biological functions of FPs are more diverse [10,11].

Using laser scanning confocal microscopy approach, spectral properties and localization of FPs in some reef-building corals (Faviina and Meandriina) were studied [12]. It was found that proteins of different colors, including green-to-red photoconvertible FPs, are localized to different tissues. Unexpectedly complex spatio-temporal patterns of distribution of a red FP in the tissues of the sea anemone *Nematostella vectensis* were revealed [13]. Interesting correlations between higher brightness of red fluorescence and greater spreading distance were revealed for *Acropora millepora* larvae [14]. These studies show complex FP functioning with still unclear molecular mechanisms of regulation and biological significance.

Here, we use confocal microscopy to study distribution and molecular mobility of endogenous FPs in live tissues of four species from Anthozoa. We demonstrate that FPs can function as solid aggregates. Inspired by this finding, we designed strongly aggregating FP variants, advantageous for some practical applications as bright and early detectable labels.

## 2. Results

### 2.1. Confocal Microscopy of Coral Polyps

**Figure 1:**
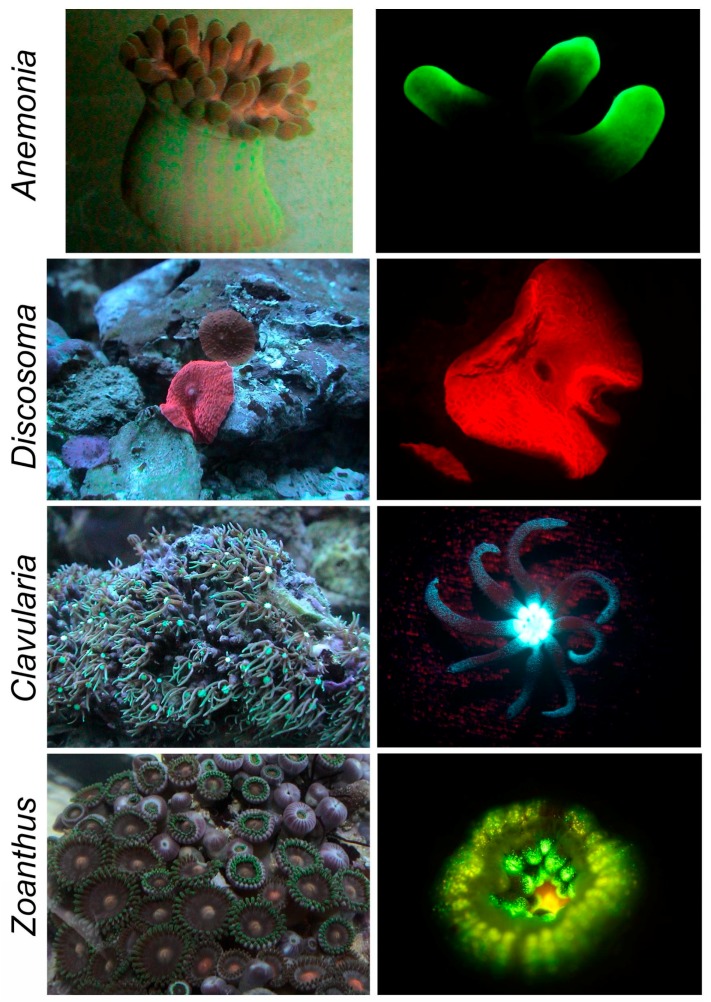
Anthozoa specimen used in this work. Left-general view, right-view of individual polyps or tentacles under a fluorescence stereomicroscope.

For our work, we chose four species from Anthozoa (available from a local aquarium store) that represent different taxonomic orders and display bright fluorescence of different colors (Figure 1): (1) sea anemone *Anemonia majano* (Hexacorallia, Actiniaria, Actiniidae); (2) mushroom coral *Discosoma* sp. (Hexacorallia, Corallimorpharia, Discosomatidae); (3) star polyp *Clavularia viridis* (Octocorallia, Alcyonacea, Clavulariidae); and (4) button polyp *Zoanthus* sp. (Hexacorallia, Zoanthidea, Zoanthidae).

Upon inspection with fluorescence stereomicroscope, single small polyps or fluorescent body parts (e.g., a tentacle) were placed in seawater onto a cover slip and immediately examined by high-resolution confocal microscopy. This enabled us to observe live coral tissues albeit not too deep from the surface. At the same time, a high thickness of the samples made it impossible to use transmitted light images for visualization of general morphology, cell nuclei and borders. Thus, in our analysis we relied on fluorescence of endogenous FPs and zooxanthellae alga (which are easily identified due to far-red chlorophyll fluorescence) only.

**Figure 2:**
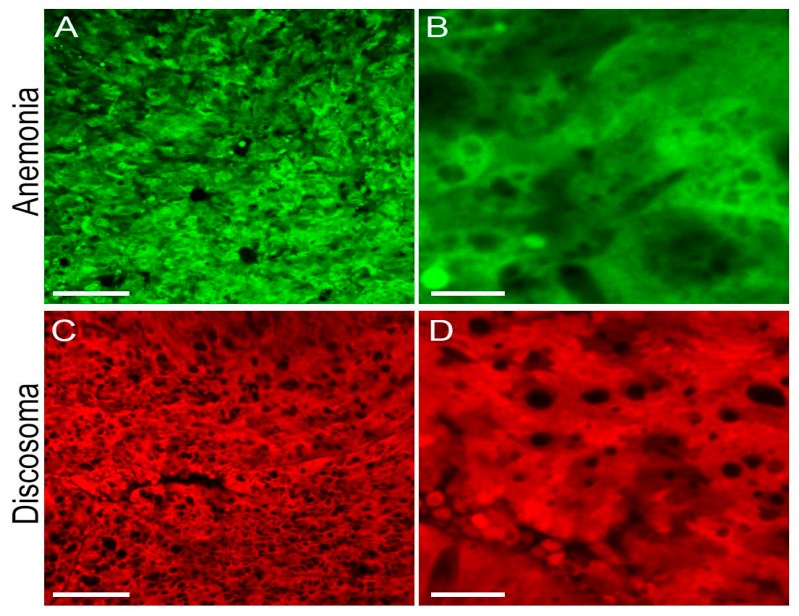
Confocal microscopy of live coral tissues. (**A**,**B**) Tip of the tentacle of *Anemonia majano*, excitation 488 nm, detection 500–540 nm (green FP); (**C**,**D**) Surface of *Discosoma* sp., excitation 543 nm, detection 560–620 nm (red FP). Scale bars: (**A**,**C**), −50 µm; (**B**), −5 µm; (**D**), −10 µm.

In the *Anemonia* specimen under study, green fluorescence was concentrated at the tentacle tips, oral disc and strips along the body. Confocal microscopy of the tentacles showed an evenly distributed green signal in the ectoderm cells (Figure 2A,B). Emission spectrum measured by the “lambda scan” regime of the microscope had a maximum at about 520 nm. In the *Discosoma* specimen nearly all ectoderm cells showed bright uniformly distributed red fluorescence with emission max at about 590 nm (Figure 2C,D).

**Figure 3:**
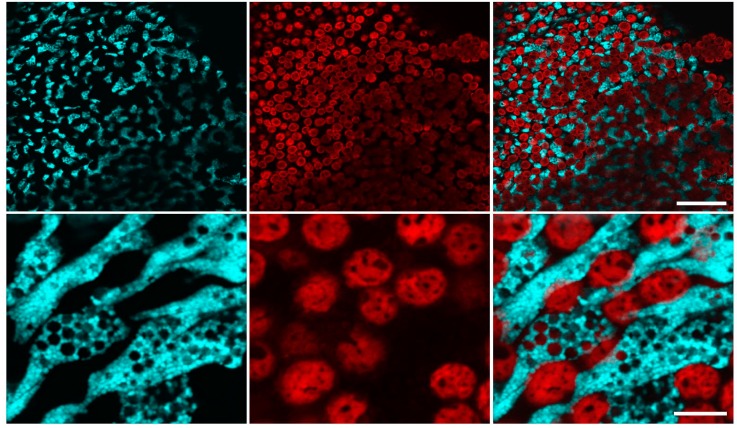
Confocal microscopy of live *Clavularia* tentacle tissues. Left, Cyan fluorescence (excitation 458 nm, detection 470–535 nm); middle, red fluorescence (excitation 543 nm, detection 570–670 nm). Right panel—overlay; Scale bars: upper row, −50 µm; bottom row, −10 µm.

*Clavularia* polyps fluoresced cyan (emission max at ~485 nm) at the oral disc and tentacle tips. Detailed inspection by confocal microscopy demonstrated that in this organism fluorescence is characteristic for endoderm cells located next to zooxanthellae alga, the latter easily identifiable due to far-red chlorophyll fluorescence (Figure 3). Intracellular distribution of the cyan signal was unusual: most of the cell volume was occupied by numerous small vesicles of about 0.7–1 µm in diameter.

**Figure 4:**
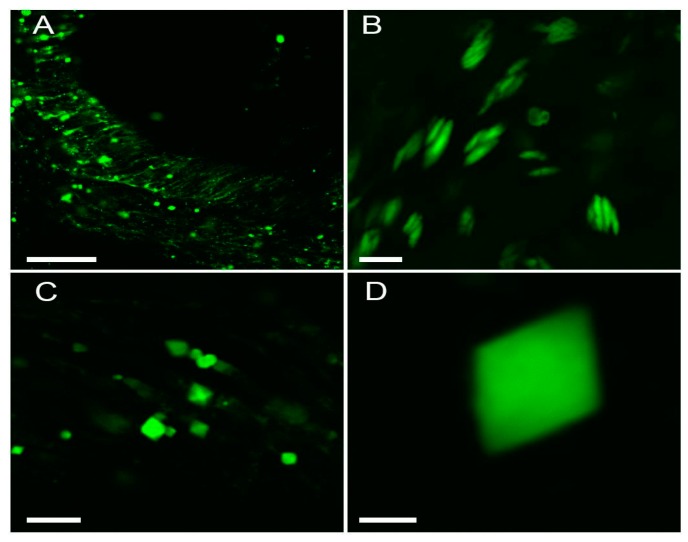
Confocal microscopy of live *Zoanthus* tentacle tissues. Green fluorescence images (excitation 488 nm, detection 500–535 nm) of representative structures (cells with diffuse signal, aggregates and crystals) at different zoom magnification are shown. Scale bars: (**A**), −50 µm; (**B**) and (**C**), −10 µm; (**D**), −2 µm.

*Zoanthus* polyp demonstrated bright green tentacle tips and a red oral disc. We studied tentacles with confocal microscopy and found green cells with long processes sparsely distributed in ectoderm (Figure 4A). Interestingly, we also observed spindle-shaped 5–10-µm-long green fluorescent granules (Figure 4B). The signal from the granules was extremely bright, roughly two orders of magnitude higher than that in a regular transient transfection of mammalian cells with e.g., an enhanced GFP (EGFP-C1) vector. In some cases, lozenge-shaped structures were detected, suggesting crystallization of the green FP within the coral cells (Figure 4C,D).

### 2.2. Fluorescence Recovery after Photobleaching (FRAP) of Endogenous of Fluorescent Proteins (FPs)

**Figure 5:**
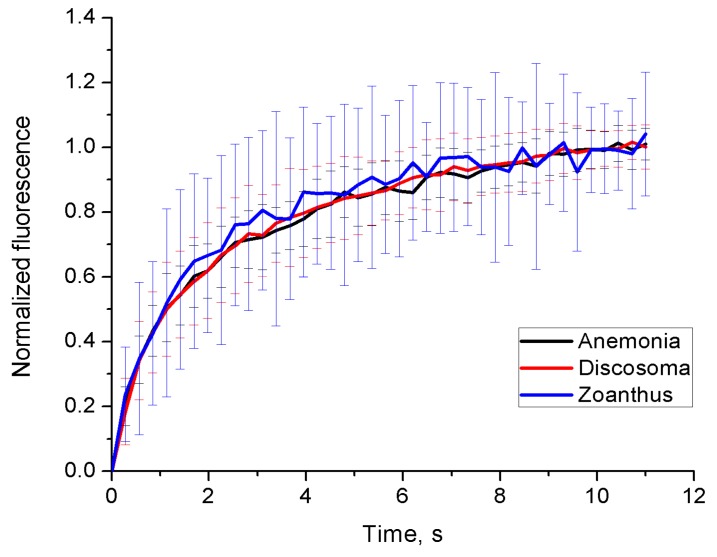
Fluorescence recovery after photobleaching (FRAP) analysis of soluble fluorescent proteins (FPs) in *Anemonia*, *Discosoma* and *Zoanthus* tissues. Graphs (mean and s.d., *n* = 30–50 cells) show recovery of fluorescence after photobleaching at a single point within cytoplasm. A full recovery of the fluorescence signal (with correction to overall partial bleaching) was observed in all these experiments.

Next, we studied dynamics of endogenous FPs using the standard fluorescence recovery after photobleaching technique. For FPs with a diffuse intracellular distribution—green FP in *Anemonia*, red FP in *Discosoma*, and green FP in *Zoanthus*—a fast and full recovery within the bleached area was observed. Notably, all three FPs showed practically indistinguishable FRAP curves (Figure 5).

**Figure 6:**
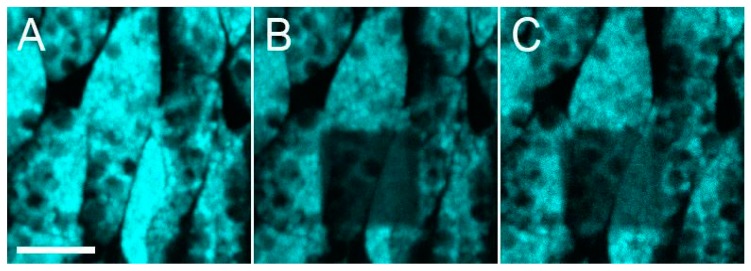
Low mobility of cyan FP-containing vesicles in *Clavularia* tissues. Cyan fluorescence before photobleaching (**A**); just after photobleaching in a square region (**B**); and 20 min after photobleaching (**C**). Scale bar, −10 µm.

For the *Clavularia* cyan FP, neither exchange between vesicles nor long-range movement of the vesicles within the cytoplasm was observed during the 20-min period after photobleaching (Figure 6). At the same time, small size of the vesicles precluded measurements of the intra-vesicle mobility of the protein.

**Figure 7:**
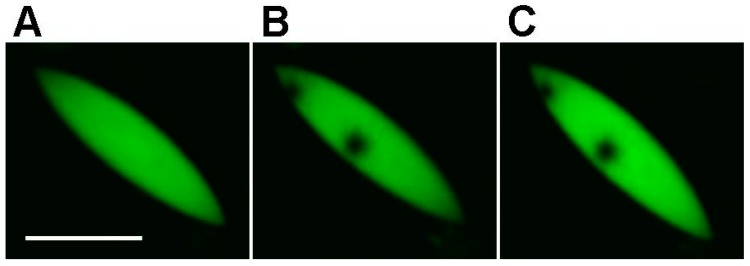
FRAP analysis of a green FP granule in *Zoanthus* tissues. (**A**) Before bleaching; (**B**) immediately after bleaching by 488 nm laser at the two points; (**C**) in 30 min after bleaching. Scale bar −10 µm.

FRAP experiments on the green granules and crystals in *Zoanthus* demonstrated complete immobility of FPs in these structures. Even after 30 min after bleaching, the shape and intensity of the bleached region remained unchanged (Figure 7).

### 2.3. Construction of Strongly Aggregating FPs

Our finding that FPs can function in nature as micron-sized granules encouraged us to develop artificial FP constructs with a strong tendency to aggregate. We reasoned that bright aggregates might be advantageous for tasks such as early detection of weak FP expression and visualization of local translation foci.

**Figure 8:**
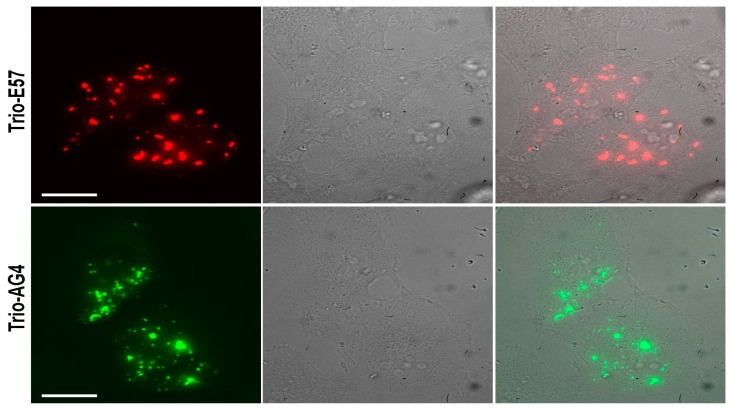
Aggregation of Trio-E57 (upper row) and Trio-AG4 (bottom row) in HeLa Kyoto cells. Left—fluorescence in red or green channels, middle—transmitted light, right—overlay. Scale bars, −20 µm.

**Figure 9:**
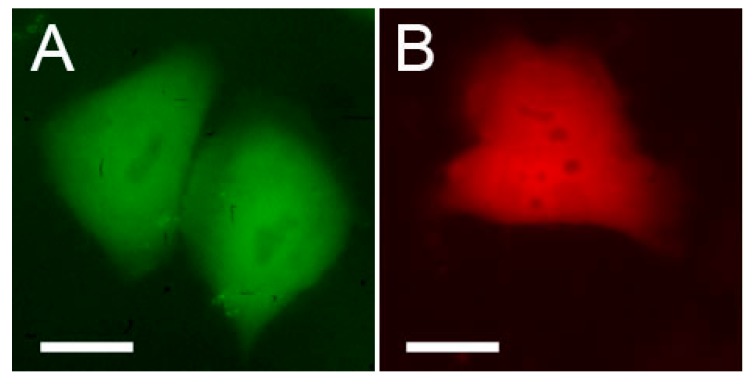
Even cytoplasmic distribution of expressing single-copy green FP AG4 (**A**) and four tandem copies of red FP E57 in HeLa Kyoto cells (**B**). Scale bar, −20 µm.

We hypothesized that a fusion consisting of three tandem copies of a tetrameric FP should strongly aggregate due to formation of intermolecular tetramers connected into an intricate network. We constructed such “trio” variants for two variants of tetrameric DsRed from *Discosoma:* red E57 and green AG4 [15]. Each vector encoded three identical copies of a complete FP sequence connected by short amino acid linkers under control of a cytomegalovirus (CMV) promoter. These vectors were transiently transfected in HEK293T cells and analyzed by fluorescence microscopy 24–48 h after transfection. Trio-E57 and Trio-AG4 formed multiple very bright spots with almost no signal between them (Figure 8). We also analyzed regular “single-unit” AG4 as well as four tandem copies of E57. Both proteins showed diffuse cytoplasmic staining with no aggregates (Figure 9).

**Figure 10:**
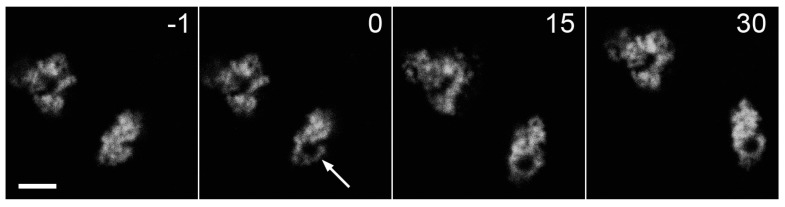
FRAP analysis of Trio-AG4 aggregates in HeLa Kyoto cells. Fluorescence was bleached by 488 nm laser at the point designated by arrow. Numbers indicate time in minutes. Scale bar, −5 µm.

FRAP analysis showed no detectable exchange of Trio-AG4 between granules during 30-min observation (Figure 10). Thus, as expected, it represents immobile aggregates in a solid-like state.

**Figure 11:**
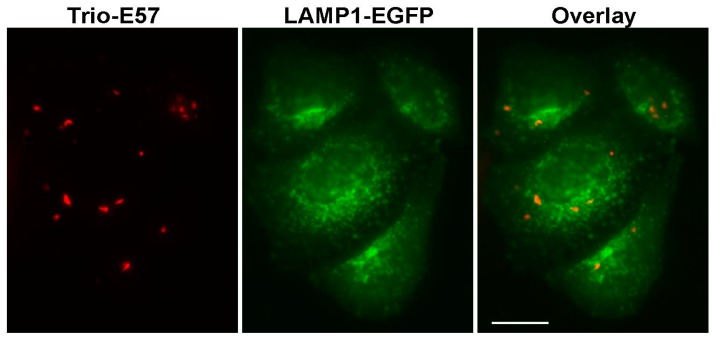
Trio-E57 aggregates and lysosomes. Wide-field fluorescence microscopy of HeLa Kyoto cells coexpressing Trio-E57 and lysosomal marker LAMP1-EGFP in red (left) and green (middle) channels. Right panel—overlay; scale bar, −20 µm.

Next we tested whether said aggregates are associated with lysosomes as previously demonstrated for some FPs [16]. To this end, we coexpressed the aggregating Trio-E57 with lysosomal marker lysosomal-associated membrane protein 1 (LAMP1-EGFP) [17]. No significant colocalization of the green and red signals was observed (Figure 11). Moreover, time-lapse imaging showed that green lysosomes undergo fast movement, whereas red Trio-E57 aggregates are practically immobile (Supplementary Movie S1).

**Figure 12:**
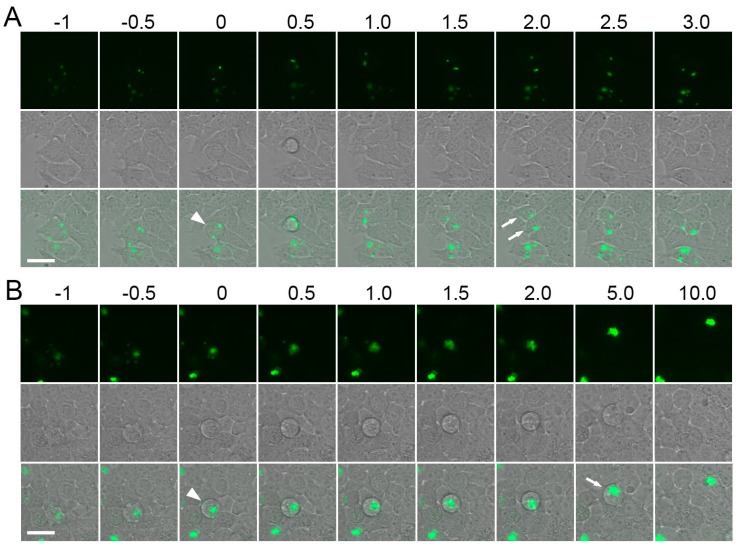
Mitosis in HEK293T cells expressing Trio-AG4. Typical examples of normal (**A**) and abortive (**B**) mitosis are shown. Numbers above the images designate relative time in hours. Upper rows—green fluorescence, middle rows—transmitted light images, bottom rows—overlay (brightness of green signal was enhanced on overlay for clarity). Formation of a rounded mitotic cell carrying fluorescent aggregates is pointed by arrowheads (time 0 h). Note that in (**A**) the cell divided into two daughter cells (two arrows, time 2.0 h), whereas in (**B**) the cell remained rounded for a long time (arrow, time 5.0 h) and did not undergo division. Scale bars, −30 µm.

We further tested possible toxicity of aggregates for mammalian cells by time-lapse imaging to follow events of cell division and death in the Trio-AG4-expressing cells. We found that cells with small aggregates undergo mitosis normally (Figure 12A). At the same time, large aggregates (usually formed as a fusion of several small foci) can result in abortive mitosis and cell death (Figure 12B). We concluded that Trio-AG4 is suitable for short-term expression at low level, but can produce significant toxicity when abundant.

**Figure 13:**
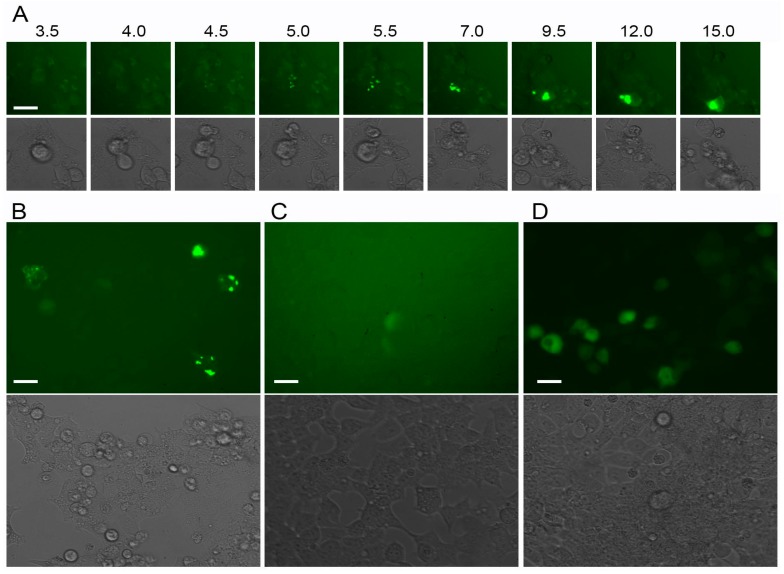
Expression of fluorescent proteins Trio-AG4 or mNeonGreen under control of H1 promoter in HEK293T cells. (**A**) Time-lapse microscopy of cell expressing Trio-AG4. Numbers above the images designate time (in hours) post transfection. Note that fluorescent spots can be reliably detected 4 h post transfection; (**B**) cells expressing Trio-AG4 15 h post transfection; (**C**,**D**) cells expressing mNeonGreen 15 h (**C**) and 72 h (**D**) post transfection. In each panel, upper images—green fluorescence, bottom images—corresponding transmitted light photos. In (**A**), fluorescence images were taken under the same settings; in (**B**–**D**)—under different settings. Scale bars, −30 µm.

To check for the potential advantages of aggregating tag for early detection of weak gene expression, we cloned Trio-AG4 under control of histone H1 promoter. This promoter is active in the S-phase of the cell cycle only; it is considered to be a weak promoter (a high expression level of H1 and other histones is achieved due to amplification of the histone genes in genome rather than increased promoter efficiency) [18]. As a control, one of the brightest green FPs available, mNeonGreen, was used under control of the same promoter. Time lapse imaging showed appearance of green spots of Trio-AG4 as early as 4–6 h after transfection (Figure 13A); many brightly fluorescent cells were observed 15 h post transfection (Figure 13B). In contrast, a few mNeonGreen-expressing cells with very weak signal (just above background) were detectable 15 h post transfection (Figure 13C). Only additional 2 days of cell growth ensured bright signal from mNeonGreen under control of H1 promoter (Figure 13D). Thus, we concluded that Trio-AG4 strongly outperforms mNeonGreen in this model.

Then, we used flow cytometry to compare Trio-AG4 and mNeonGreen in more detail. HEK293T cells were transiently transfected in parallel with vectors encoding Trio-AG4 or mNeonGreen under control of either CMV or H1 promoters. This side-by-side comparison showed the following (Table 1). For CMV-driven expression, mNeonGreen gave a much brighter signal than Trio-AG4, especially on the first day after transfection. In contrast, in the case of weak H1 promoter Trio-AG4 ensured about a two-fold higher level of fluorescence compared to mNeonGreen. As integral signal intensity in flow cytometry is practically independent of intracellular localization (i.e., aggregated FP spots would give the same signal as corresponding amounts of soluble FP), we concluded that enhanced detection of Trio-AG4 in the H1 promoter samples is probably due to increased stability of FP in the aggregated state against protein degradation.

## 3. Discussion

Confocal microscopy of endogenous FPs in vivo can help to unravel their diverse biological functions in anthozoans and other organisms. To the best of our knowledge, we for the first time applied FRAP to study the molecular mobility of FPs in live tissues of Anthozoa specimens. We observed essentially identical fast diffusion of soluble FPs in *Anemonia*, *Discosoma* and *Zoanthus*. Considering the rather distant relationship between these species (different orders) and color variations (green and red FPs), our finding suggests that many natural FPs are freely diffusible cytoplasmic proteins, probably with no association with other intracellular structures or proteins.

At the same time, we found two cases of strictly immobilized FPs. Although we did not perform additional staining for membranes, we believe that in *Clavularia* cyan FP is probably localized to vesicles. First, their shape was always rounded (in contrast to aggregates and crystals in *Zoanthus* cells). Even more importantly, the cloned cyan FP from *Clavularia* contains an N-terminal signal peptide [2] that probably directs its synthesis into the specialized vesicles observed here. *Zoanthus* shows another striking example of intracellular FP distribution as aggregates and crystals of several microns in size. These structures contain FP in a “solid” state with no internal mobility of protein molecules.

Examples of protein functioning as aggregates or crystals are rare and mostly associated with pathological processes. Here we showed that FPs can naturally occur as large aggregates or crystals. Many wild-type FPs from Anthozoa species were found to strongly aggregate in vitro [19,20]. Also, some engineered variants of FPs that spontaneously form crystals in mammalian cells were described [21]. This suggests that FP aggregation may be a rather general phenomenon characteristic not only for *Zoanthus* species. Possibly, the presence of FPs in aggregates, crystals or within vesicles provides a way to accumulate a large amount of FPs at the levels unachievable for a soluble protein in cytoplasm. Also, light scattering on FP granulas was proposed to have a role in decrease of heating of deep coral tissues [22].

Compared to the diffuse fluorescent signal, localized aggregates are better detectable and provide additional ways for structure-functional characterization in recombinant models. For example, the nanobody-mediated accumulation of a GFP-tagged protein partner in an intracellular locus (e.g., a spot in the nucleus) provides an opportunity to study its interaction with another red FP (RFP)-tagged partner through colocalization in that spot [23]. Recently, a spectacular method based on reversible formation of liquid-phase FP-containing droplets in the cytoplasm was developed to monitor protein–protein interactions [24]. Here, we designed strongly aggregated versions of tetrameric green and red FPs by concatenating their three tandem copies. The formed intracellular bright foci are excellently visible compared to regular diffusely distributed FP and thus enhance early detection of weak promoters. Increased brightness of Trio-E57 and Trio-AG4 probably results from a combination of several factors: presence of multiple FP copies instead of one, concentration of the fluorescence signal within small foci which are easier to detect against background, and increased stability against degradation by intracellular protein turnover machinery. High brightness and immobility of FP aggregates can be useful for other tasks such as visualization of spatio-temporal patterns of local translation. At the same time, larger aggregates can induce significant cytostatic and cytotoxic effects. Thus, these tags appear to be advantageous only for models with short-term low-level expression.

## 4. Materials and Methods

### 4.1. Confocal Microscopy

Anthozoa specimens were obtained at the local aquarium store (Available online: www.aqualogo.ru). Single polyps or small pieces of tissues were placed on a cover glass in seawater and immediately used for microscopy. Fluorescence stereomicroscope SZX12 (Olympus, Tokyo, Japan) with a color CCD camera was used to get a general view of the specimens and tissues. The following filters were used: excitation 400–410 nm, detection >440 nm (blue-cyan channel); excitation 450–480 nm, detection >520 nm (green channel); excitation 435–455 nm, detection >600 nm (red channel).

Laser scanning confocal microscopy was performed using an inverted microscope DMIRE2 TCS SP2 (Leica, Wetzlar, Germany) with an HCX PL APO lbd.BL 63× 1.4NA oil objective. Ar (458 and 488 nm lines) and HeNe (543 nm) lasers were used to excite cyan, green and red fluorescence, respectively. FRAP analysis of fast-diffusing FPs in *Anemonia*, *Discosoma* and *Zoanthus* tissues was performed by taking 40 consecutive images of 25 µm × 25 µm area (64 × 64 pixels, 3.5 fps) immediately after local fluorescence bleaching for 0.25 s with 100% 488 nm (for the green FPs) or 543 nm (for the red FP) at a single point within a cell. FRAP analysis of vesicles and aggregates (in *Clavularia* and *Zoanthus* tissues, respectively) was performed by bleaching of a region or a point with 100% 458 or 488 nm laser, followed by a 30-min observation period. Manual correction of focus and position of the area of interest was used to compensate for movements of live tissues.

### 4.2. Cloning

Standard PCR and cloning methods were used to construct “Trio” versions of E57 and AG4 proteins [10]. As a result, each vector encoded three identical tandem copies of either E57 or AG4 connected by linkers RSPG (between first and second FP) and RTRPVAT (between the second and third FP) under control of the CMV promoter in the standard C-vector backbone. Linkers in the variant of four tandem copies of E57 were RTRPVAT and RSPG.

A 400-bp fragment of histone H1 promoter [13] was amplified on a template of HeLa genomic DNA using primers H1-for 5′-ATATATTAATGATGCCCTCAGCCCAATGGATTC and H1-rev 5′-AAATACCGGTGGGGTGGCTGTCTCGCCAGGAGC (Ase I and Age I restriction sites are underlined). This fragment was cloned using Ase I and Age I in Trio-AG4 and mNeonGreen vectors instead of a CMV promoter.

### 4.3. Mammalian Cells

HEK293T and HeLa Kyoto cells were grown under standard conditions and transfected with FuGene 6 reagent (Promega, Fitchburg, WI, USA) in accordance to the manufacturer’s protocol.

### 4.4. Wide Field Fluorescence Microscopy

For wide-field fluorescence microscopy of mammalian cells, an AF6000 LX inverted microscope (Leica, Wetzlar, Germany) with an HCX PL APO lbd. BL 63× 1.4NA oil objective and a CoolSNAP HQ CCD camera (Photometrics, Tucson, AZ, USA) was used. Green and red fluorescence were acquired using standard filter cubes: GFP (excitation 450–490 nm, emission 500–550 nm) and TX2 (excitation 540–580 nm, emission 610–680 nm). Prolonged time-lapse imaging was performed at 37 °C in a 4-(2-hydroxyethyl)-1-piperazineethanesulfonic acid (HEPES)-buffered imaging medium.

### 4.5. Flow Cytometry

Flow cytometry analysis of live cells was performed using Cytomics FC500 (Beckman Coulter, Indianapolis, IN, USA). Fluorescence was excited with a 488-nm laser and detected at 510–540 nm using the same settings for all cell samples.

## Figures and Tables

**Table 1:** Flow cytometry analysis of HEK293T cells transiently expressing Trio-AG4 or mNeonGreen fluorescent proteins under control of CMV or H1 gene promoters.

Promoter	Fluorescent Protein	Mean (Median) Green Fluorescence Intensity, Arbitrary Units (a.u.)		
		24 h	48 h	72 h
CMV	Trio-AG4	86 (47)	458 (209)	450 (147)
	mNeonGreen	1088 (259)	800 (146)	806 (110)
H1	Trio-AG4	57 (20)	95 (40)	117 (47)
	mNeonGreen	32 (20)	47 (26)	50 (29)

## Supplementary Materials

Supplementary materials can be found at www.mdpi.com/1422-0067/18/7/1503/s1.

## Abbreviations

GFP	Green fluorescent protein
FP	Fluorescent protein
FRAP	Fluorescence recovery after photobleaching
RFP	Red fluorescent protein
