# Supplementary material for: Functioning of Fluorescent Proteins in Aggregates in Anthozoa Species and in Recombinant Artificial Models
Source: Int J Mol Sci. 2017 Jul 12;18(7):1503. doi: 10.3390/ijms18071503 (PMC5535993; doi:10.3390/ijms18071503)
Supplement: Supplementary file 1 [file ijms-18-01503-s001.zip › SupplMovie1 capture.pdf]

Supplementary Movie 1. Time-lapse fluorescence microscopy of Trio-E57 aggregates (red) and lysosomes (LAMP1-EGFP, green) in HeLa Kyoto cells. Movie shows 135-s time interval, 40 frames.
